# *Mycobacterium abscessus* induces immune suppression in cystic fibrosis macrophages

**DOI:** 10.3389/fcimb.2026.1903803

**Published:** 2026-10-27

**Authors:** Preston J. Hill, Christopher J. Miller, Raul Rascon, Gillian Clary, Ayantu W. Idosa, Mikhail A. Gavrilin, Karen S. McCoy, Amal O. Amer, Santiago Partida-Sanchez, Benjamin T. Kopp, Luanne Hall-Stoodley

**Affiliations:** 1 Department of Microbial Infection & Immunity, The Ohio State University, Columbus, OH, United States; 2 Center for Microbe and Immunity Research, Abigail Wexner Research Institute at Nationwide Children’s Hospital, Columbus, OH, United States; 3 Department of Internal Medicine, College of Medicine, The Ohio State University, Columbus, OH, United States; 4 Division of Pulmonary Medicine, Nationwide Children’s Hospital, Columbus, OH, United States; 5 Center for Perinatal Research, Abigail Wexner Research Institute at Nationwide Children’s Hospital, Columbus, OH, United States; 6 Infectious Disease Institute, The Ohio State University, Columbus, OH, United States

**Keywords:** cystic fibrosis, infection - immunology, macrophages, *Mycobacterium abscessus* (*MAB*), nontuberculous mycobacteria

## Abstract

**Introduction:**

People with cystic fibrosis (pwCF) are highly susceptible to chronic lung infections with *Mycobacterium abscessus* (*MAB*), a nontuberculous mycobacterium (NTM) that is intrinsically resistant to antibiotics. While macrophage dysfunction is recognized in CF, responses to *MAB* in primary human macrophages remain poorly defined.

**Methods:**

We investigated the innate immune response to *MAB* using peripheral blood monocytes and monocyte-derived macrophages from pwCF and healthy donors. Macrophages were infected with opsonized smooth (*MAS*) or rough (*MAR*) isolates of *MAB* morphotypes at low multiplicity of infection. After removal of extracellular bacteria, intracellular burden, oxidative burst, NOX2 activation, and cytokine production were assessed at 2- and 24-hours post-infection.

**Results:**

*MAB* uptake was comparable across all groups and non-CF macrophages showed no difference in intracellular *MAR* or *MAS* at 24h. In contrast, CF macrophages harbored significantly more intracellular *MAR* at 24 hours, a phenotype recapitulated by CFTR knockdown in non-CF macrophages. *MAB*-infected CF macrophages demonstrated a significantly reduced oxidative burst and diminished NOX2 p47phox phosphorylation. Non-CF macrophages treated with CFTR inhibitor mimicked the reduced oxidative burst. CF macrophages also exhibited an altered cytokine response with significantly elevated IL-8 but decreased IL-1β relative to healthy macrophages. Notably, *MAR*-infected CF macrophages secreted significantly less TNF-α compared with non-CF macrophages and exhibited markedly reduced apoptosis compared with *MAR*-infected non-CF and *MAS*-infected CF macrophages.

**Discussion:**

These findings demonstrate that CF macrophages are intrinsically impaired in their ability to control the virulent rough *MAB* morphotype, highlighting a potential mechanism for persistent NTM infection in pwCF and underscoring the need for host-directed therapies.

## Introduction

Cystic fibrosis (CF) is caused by mutations in the cystic fibrosis transmembrane conductance regulator (*CFTR*) gene, resulting in diminished or absent CFTR ion channel function in multiple cell types. Abnormal CFTR in airway epithelial cells leads to the accumulation of viscous mucus providing a microenvironment that harbors bacteria and promotes increased susceptibility to bacterial infections (Collawn and Matalon, 2014). Studies indicate that defective CFTR function in monocytes and macrophages also plays a key role in pulmonary infections in people with CF (pwCF) contributing to the progression of chronic lung disease by failing to clear bacteria and triggering excessive inflammatory responses (Bruscia et al., 2009; Bruscia and Bonfield, 2016; Assani et al., 2017; Jaganathan et al., 2022). For pwCF with variants sensitive to elexacaftor/tezacaftor/ivacaftor (ETI), CFTR modulator therapy has decreased pulmonary exacerbations and increased lung function. However, studies suggest that CF immune cell function is not fully restored with CFTR modulator treatment (Jaganathan et al., 2022; Kopp et al., 2020; Zhang et al., 2023).

Worldwide, pulmonary infections due to nontuberculous mycobacteria (NTM) have become increasingly common (Ratnatunga et al., 2020) and pwCF show an exceptionally high susceptibility with an approximately 1,000-fold greater prevalence of NTM based on culture data (Catherinot et al., 2013; Floto et al., 2016; Adjemian et al., 2018; Martiniano et al., 2022). *Mycobacterium abscessus* (*MAB*) has emerged as one of the most resistant NTM to antibiotic therapy linked to persistent infection in people with structural pulmonary diseases including chronic obstructive pulmonary disease (COPD) and CF (Daniel-Wayman et al., 2019; Swenson et al., 2018; Cystic Fibrosis Foundation Patient Registry Highlights, 2022). *MAB* can lead to implacable infections, severe lung disease with end-stage bronchiectasis, hastened lung function decline, and poor prognosis in pwCF with diminished long-term survival relative to *Mycobacterium avium* (Martiniano et al., 2022; Floto et al., 2016; Catherinot et al., 2013; Daniel-Wayman et al., 2019; Swenson et al., 2018). Despite protracted combinatorial antibiotic therapy, *MAB* infection may not be eradicated, and adverse therapy effects can further limit treatment options, threatening an improved quality of life in pwCF.

*MAB* presents as a smooth morphotype (*MAS*) that expresses high levels of cell wall glycopeptidolipids (GPL) or rough (*MAR*) that expresses limited GPL. *MAB* morphotypes have distinct effects on macrophage bactericidal activity and invasiveness (Rhoades et al., 2009; Clary et al., 2018). *MAR* exhibited greater virulence in a longitudinal study (Catherinot et al., 2009) and significantly lower cure rates than *MAS*, reinforcing its reputation as a highly pathogenic *MAB* variant (Koh et al., 2017; Hedin et al., 2023). In previous studies we showed that each *MAB* morphotype is equally taken up by human THP-1-derived macrophages where bacteria survived intracellularly for up to 48 hours post-infection, including in macrophages treated with antibiotics (Clary et al., 2018). Recent studies indicate that recruited monocyte-derived macrophages are significantly increased in pwCF and play a central role in signaling and recruiting innate and adaptive immune cells in addition to alveolar macrophages in the lung (Schupp et al., 2020). In the present study primary monocytes and differentiated monocyte- derived macrophages (MDM) from peripheral blood mononuclear cells from CF and healthy non-CF donors were investigated as a relevant model to better understand the role of these cells in early *MAB* infection. CF macrophages exhibited a reduced respiratory burst associated with impaired bacterial killing and a distinct cytokine inflammatory profile relative to healthy macrophages. We provide evidence that CFTR dysfunction in primary human monocyte-derived macrophages impaired functional responses to *MAB* infection and the first evidence that CF macrophages infected with rough *MAB* showed heightened deficiencies relative to healthy macrophages as well as to CF macrophages infected with the smooth morphotype. The difference in microbial killing between *MAR* and *MAS* suggests unique pathogen-host interactions that could inform immune-targeted therapies to enhance bacterial clearance and slow lung function decline (Zhang et al., 2023; Catherinot et al., 2013; Daniel-Wayman et al., 2019; Swenson et al., 2018).

## Methods

### Ethics statement

All human subjects were recruited as approved by the Institutional Review Board of Nationwide Children’s Hospital (IRB16-01020) and the Ohio State Biomedical Sciences IRB (IRB 2016H0141). All subjects underwent written consent for the procedures; all adult subjects provided informed consent, and a parent or guardian of any child participant with CF provided informed consent on their behalf along with written assent from children. All non-CF blood donors were adults and provided written informed consent to participate in this study.

### Bacterial strains and culture

*M. abscessus sensu stricto* is the most prevalent strain and most refractory to antibiotic therapy (Adjemian et al., 2018; Martiniano et al., 2022). The *MAB* Type strain 19977, containing a mixture of both *MAS* and *MAR* morphotypes, was obtained from the American Type Culture Collection (Manassas, VA) and isogenic colony variants were isolated and cultured as described (Clary et al., 2018). Briefly bacteria were streaked from isolated *MAS* and *MAR* frozen stocks and cultured for 5–7 days at 37 °C/5% CO_2_ on 7H10 agar containing 10% oleic acid-albumin dextrose-catalase (OADC).

To ensure that *MAS* and *MAR* suspensions for infection were not aggregated, isolated morphotypic colony variants were placed in Eppendorf tubes, each containing 1 mL of 7H9 broth–OADC–0.5% Tween 80, pulse vortexed with glass beads, and allowed to settle for 30 min, after which 600 mL of the supernatant was transferred to a second tube. After 10 min, 300 mL was transferred from each tube to a new tube. Bacteria were transferred to a Petroff-Hausser chamber and counted as described (Schlesinger, 1993; Hall-Stoodley et al., 2006; Clary et al., 2018) resulting in a >90-95% single cell bacterial suspension. Suspensions were checked microscopically to ensure a single cell suspension for infection and to assess colony morphotypes post-infection for enumerating colony forming units (CFU).

### Human monocyte isolation, macrophage differentiation, and bacterial infection

Heparinized blood was obtained from 35 CF and 25 healthy control individuals. A history of chronic immunosuppression including chronic steroid use or history of transplantation was excluded in CF donors, but chronic inhaled antibiotic and CFTR modulator use was allowed. Peripheral blood mononuclear cells (PBMCs) were separated from whole blood using lymphocyte separation medium (25-072-CV; Corning) and isolated monocytes were resuspended in RPMI 1640 (22400-089; Life Technologies) supplemented with 10% human AB serum (Sigma #H4522-100ML) and cultured at 37 °C to differentiate into macrophages as described with modification (Assani et al., 2017). Briefly, for monocyte assays, monocytes were isolated by adherence for 2h, washed to remove nonadherent cells, and resuspended in RPMI 1640/10% human AB serum, placed on ice for 10 minutes, washed and enumerated for assays. For monocyte-derived macrophages, monocytes were cultured in Teflon wells for 6 days and plated in 24 well tissue-culture plates overnight to differentiate macrophages. Following attachment, macrophages were washed to remove unattached cells and counted using a hemocytometer to determine the appropriate multiplicity of infection (MOI). Human non-CF and CF- macrophages were infected with a single-cell suspension (>95% single cells) of *MAS* or *MAR* obtained by direct counting of bacteria in a Petroff-Hausser chamber as previously described (Clary et al., 2018). Immediately prior to infection, bacteria were opsonized with AB serum for 30 minutes and macrophages were infected for 2h at 2.5:1 MOI. Notably, these steps circumvent bacterial aggregation that can occur in tissue culture medium without tween. At 2h, infected monolayers were rinsed vigorously to remove extracellular bacteria and either lysed and plated on 7H10 agar to enumerate CFUs at this timepoint to determine uptake or repleted with 10% AB human serum and incubated further for 24h.

The THP-1 monocytic line (ATCC #) was grown in 10% FBS (Thermo Scientific) in RPMI 1640. THP-1 cells were treated with 200 nM of Phorbol 12-myristate 13-acetate (PMA) (Sigma-Aldrich, #P8139-1MG) and 30 ng/mL GM-CSF (415-ML-050; R&D Systems) to differentiate cells into macrophages (Daigneault et al., 2010). Media were replenished with 30 ng/ml GM-CSF and the THP-1–derived macrophages were allowed to mature 5 days before experiments, plated overnight, and infected with *MAS* or *MAR* as above at a MOI of 25:1 (Gavrilin et al., 2021; Clary et al., 2018). CFUs were enumerated at 2 and 24h post-infection.

### CRISPR/Cas9 THP-1 cells

Lentivirus clones of stable THP-1 cells expressing Cas9-mCherry CRISPR constructs were generated as described (Gavrilin et al., 2021) with the following modifications: sgCFTR-EGFP virus was used to make double positive cells stably expressing Cas9 and CFTR guide RNA. Briefly, the pFUCas9-mCherry plasmid was used for Cas9 expression and the doxycycline hyclate-inducible pFgH1t_UTG plasmid expressing the EGFP marker (both gifts from Dr. S. Masters, Walter and Eliza Hall Institute of Medical Research, Australia) (Baker and Masters, 2018) was used for CRISPR constructs. Small guide RNAs against CFTR was designed using CHOPCHOP v2; sgCFTR (minus strand) 5’ – TCTGTATCTATATTCATCAT –3’. Sense and antisense guide RNA oligonucleotides were brought to 100 μM, annealed and the plasmid was digested with the *BsmBI* restriction enzyme (New England Biolabs Inc), run on a 1% agarose gel, excised, eluted and used for ligation of annealed sgRNA oligonucleotides. Plasmids were verified by Sanger sequencing. sgCFTR lentivirus was produced as described (Gavrilin et al., 2021), titrated, and was used for transduction of stable Cas9-mCherry THP-1 cells by spinoculation (800 x g for 90 min) using LentiBoost solutions (Sirion Biotech). After recovery, mCherry/EGFP double positive cells (>80%) were sorted by flow cytometry with FACS ARIA III (BD Biosciences) resulting in a nearly homogeneous population of Cas9-sgCFTR THP-1 cells. Cells were then induced twice with 1 μg/ml of doxycycline hyclate (Sigma) for 72 h and CFTR knockdown (KD) was verified by immunoblot using CFTR 24–1 or CFTR H182 primary antibodies for detection of CFTR protein. Clonal selection of the best clone (10F8) was performed. Cells were tested for the absence of mycoplasma contamination and used for infection studies (Zakharova et al., 2010). **Supplementary Figures 1B, C** show that Clone 10F8 used for infection had the greatest reduction in CFTR compared to homogenous Cas9-sgCFTR THP-1 cells.

### Cytotoxicity and apoptosis assays

Macrophage cytotoxicity was measured using the lactate dehydrogenase (LDH) Cytotoxicity Assay Kit (Cell Signaling Technologies, Danvers, MA), a stable, soluble enzyme located in the cytosol of cells and released into the surrounding culture medium upon plasma membrane damage. After plating, supernatants from uninfected or 24h *MAS*-infected or *MAR*-infected differentiated macrophages from CF or non-CF donors were transferred to a flat bottomed 96 well plate, mixed with the reaction solution per the manufacturer’s instructions, and absorbance was measured at 490nm using a SpectraMax M3 plate reader (Molecular Devices, Sunnyvale CA). Infection-induced LDH release was calculated as % LDH = (infected sample − respective negative control)/(respective positive control − respective negative control)) * 100.

Apoptosis was measured for uninfected CF and non-CF MDM or macrophages infected with *MAS* or *MAR* using the Click-iT Plus TUNEL Assay (Invitrogen C10617, Invitrogen/Life Technologies Corp, Carlsbad, CA) according to the manufacturer’s instructions.

### Measurement of oxidative burst and reactive oxidative species and inhibition of CFTR

The oxidative burst produced by monocytes was measured by the luminol chemiluminescence assay (Assani et al., 2017). Bacteria were opsonized in suspension using 10% human AB serum in HBSS for 30 minutes at 37°C/5% CO_2_ and washed in HBSS prior to the generation of the reactive oxygen species (ROS) response by monocytes. PMA (10 ng/mL) was used as a positive agonist control for ROS generation. Monocytes (4 x 10^6^) were seeded on white, opaque, flat-bottom 96 well microtiter plates (Falcon) and opsonized bacteria were added to each well at a MOI of 10:1 in the presence of 50 mM luminol. All samples and controls were seeded in triplicate. The microtiter plate was centrifuged at 800 x g, 4°C for 3 minutes to synchronize the infection. The relative amount of generated ROS was detected over 60 minutes by measuring luminescence at 3-minute intervals on a SpectraMax M3 plate reader (Molecular Devices, Sunnyvale CA). Relative Intensity Units (RIU) were plotted against time and the Area Under the Curve (AUC) compared for statistical differences.

For macrophages, the oxidative burst was measured by a 2’,7’-dichlorofluorescein (DCF) assay (D399; Life Technologies) using relative fluorescent units (RFUs). Macrophages were adhered to black, clear flat bottom 96-well microtiter plates at 3x10^5^ cells/well in triplicate for 2h and repleted in DPBS + 10 mM HEPES + 1 mg/mL human serum albumin + 0.1% glucose. After 30-min incubation at 37 °C/5% CO_2_, 10% DCF was added to the wells for 30 min at 37 °C. PMA (200nM) was added to macrophages, and fluorescence was measured at 485 nm excitation wavelength and 515 nm emission wavelength every 3 min for 90 minutes. The relative amount of generated ROS was detected over 90 minutes by measuring fluorescence at 3-minute intervals on a SpectraMax M3 plate reader (Molecular Devices, Sunnyvale CA). Relative Intensity Units (RIU) were plotted against time, and AUC data were compared for statistical differences.

For CFTR inhibition studies, non-CF monocyte-derived macrophages were cultured as above and CFTR INH-172 (Sigma Aldrich, #C2992, St. Louis, MO) was used as a voltage-independent, selective CFTR chloride channel blocker at a concentration of 5 μM, incubated with healthy MDM for 24hrs, and measured as above.

### Immunoblotting

Human macrophages were obtained and differentiated as described above. Briefly, 4x10^6^/mL monocytes were seeded into 6 well tissue culture plates, incubated overnight at 37°C/5% CO_2_ to allow attachment, washed with RPMI to remove non-adherent cells and resuspended in RPMI + 10% human AB. Macrophages were infected at an MOI of 2.5 for 2 or 24h with opsonized *MAS* or *MAR* in RPMI + 10% human AB serum for 30 minutes at 37°C/5% CO_2_. Supernatants were removed post-infection and snap-frozen at -80°C prior to cytokine analyses. Infected macrophages were lysed in lysis buffer (10 mM Tris-HCl, Nonidet P-40 and Complete protease inhibitor (10-519-978-001; Roche Applied Science) as described (Assani et al., 2017). Briefly, the lysed suspension was transferred to a sterile Eppendorf tube, and samples were concentrated by Cytvia Nanosep 3000 MWCO centrifugal devices (Fisher Scientific, #50-197-9590), washed with sterile DPBS, and protein concentrations estimated by micro-BCA (BCA Protein Assay Kit, Pierce). Protein (15 μg) was separated by SDS-PAGE (Bolt Bis-Tris Plus 4-12% Bis-Tris 12-well gels, Invitrogen #NW04122) and transferred onto nitrocellulose membranes. Membranes were immunoblotted for phospho-p47^phox^, gifted by Jamel El- Benna (Belambri et al., 2022), phospo-p40^phox^ (#4311, Cell Signaling), total p47^phox^ (A16636, Life Technologies), total p40^phox^ (ab137691, Abcam), gp91^phox^ (SC-5827, Santa Cruz) to evaluate nicotinamide adenine dinucleotide phosphate (NADPH) oxidase (NOX) components, protein kinase C (PKC) phosphorylation p-PKC α β II (9375P, Cell Signaling), p-PKC δ (9374P; Cell Signaling), and p-PKC ζ/γ (9378P, Cell Signaling), IL-1β (SC-32294, Santa Cruz), caspase-1 (SC56036 Santa Cruz), and GAPDH (14C10, Cell Signaling Technology). Protein bands were detected with HRP-conjugated secondary antibodies and visualized using ECL reagents (RPN2106, Life Sciences). Densitometry analyses were performed by normalizing target protein bands to their respective loading control (GAPDH) using ImageJ software.

### Cytokine analyses

ELISA was performed to determine the levels of TNF-α, IL-8, IL-6, MIP-1β and IL-1β in the cell supernatants collected from *MAS* and *MAR* infected macrophages. Cell supernatants were collected and stored at −80 °C as described above. Cytokines were detected for human IL-8 using IL-8 DuoSet ELISA (R&D systems, DY208-05), human IL-6 DuoSet ELISA (R&D systems, DY206-05), human MIP-1β DuoSet ELISA (R&D systems, DY207-05), human TNF-α DuoSet ELISA (R&D systems, DY210-05), and human IL-1β DuoSet ELISA (R&D systems, DY201-05) according to the manufacturer’s protocols. Pro-inflammatory cytokine levels were determined using the standard curve samples provided in each kit.

### Statistical analysis

All statistical analyses were performed using GraphPad Prism software (version 10). Normality testing was performed when it was a prerequisite to utilize a particular statistical test. When data failed to meet the assumptions of normality, non-parametric statistical tests were used. Two sample unpaired t tests, one-way ANOVA or two-way ANOVA with multiple comparison analyses were used for comparisons of independent samples. Statistical significance was defined as p < 0.05.

## Results

### Cohort characteristics: supplementary data

The mean age of CF blood donors was 18.79 years (SD = 11.64), 39.4% were male and 75.8% were homozygous for the F508del *CFTR* variant, while 24.2% were heterozygous. Mean BMI was 21.48 (SD = 4.39) and 60.6% were on CFTR modulator therapy. Mean FEV_1_ was 92.4 (SD = 17.34, range=39-126).

### Primary differentiated human CF macrophages harbored significantly more intracellular *MAR* compared to *MAS* at 24h post-infection

Enumeration of viable bacteria by CFU at 2h post-infection (p.i.) showed no significant difference between non-CF and CF macrophages in intracellular *MAS* or *MAR* (**Figure 1A**). In contrast, at 24h p.i. CF macrophages harbored significantly more intracellular viable *MAR* compared with *MAS*-infected and *MAR*-infected healthy macrophages (p=0.027) as well as significantly more *MAR* relative to CF *MAS*-infected macrophages at 24h p.i. (p=0.0007), Tukey’s multiple comparisons test (N = 3 non-CF, N = 4 CF donors, in triplicate) (**Figure 1B**). To evaluate if the difference in *MAB* morphotype survival at 24h post-infection was CFTR dependent, differentiated THP-1 macrophages (Clary et al., 2018) were treated with the Cas9 guide RNA alone (*CFTR^+/+^*) or Cas9-mediated *CFTR* knock down (KD) that disrupted CFTR function (*CFTR^-/-^*). Differentiated THP-1-like macrophages with Cas9 only (*CFTR^+/+^*) (**Figure 1C**) showed no difference in viable *MAR* and *MAS* at 24h post-infection (p=0.5156), comparable to primary non-CF macrophages at this time point. However, Cas9-*CFTR* KD (*CFTR^-/-^*) macrophages showed a significantly higher burden of *MAR* relative to *MAS* (p=0.0020), Wilcoxon t-test, recapitulating the defect seen in primary CF macrophages (N = 4 biological replicates each, with 3 technical replicates). The uptake at 2h post-infection of *MAS* and *MAR* in Cas9 only and Cas9-*CFTR* KD was not significantly different (**Supplementary Figure 1A**).

**Figure 1:**
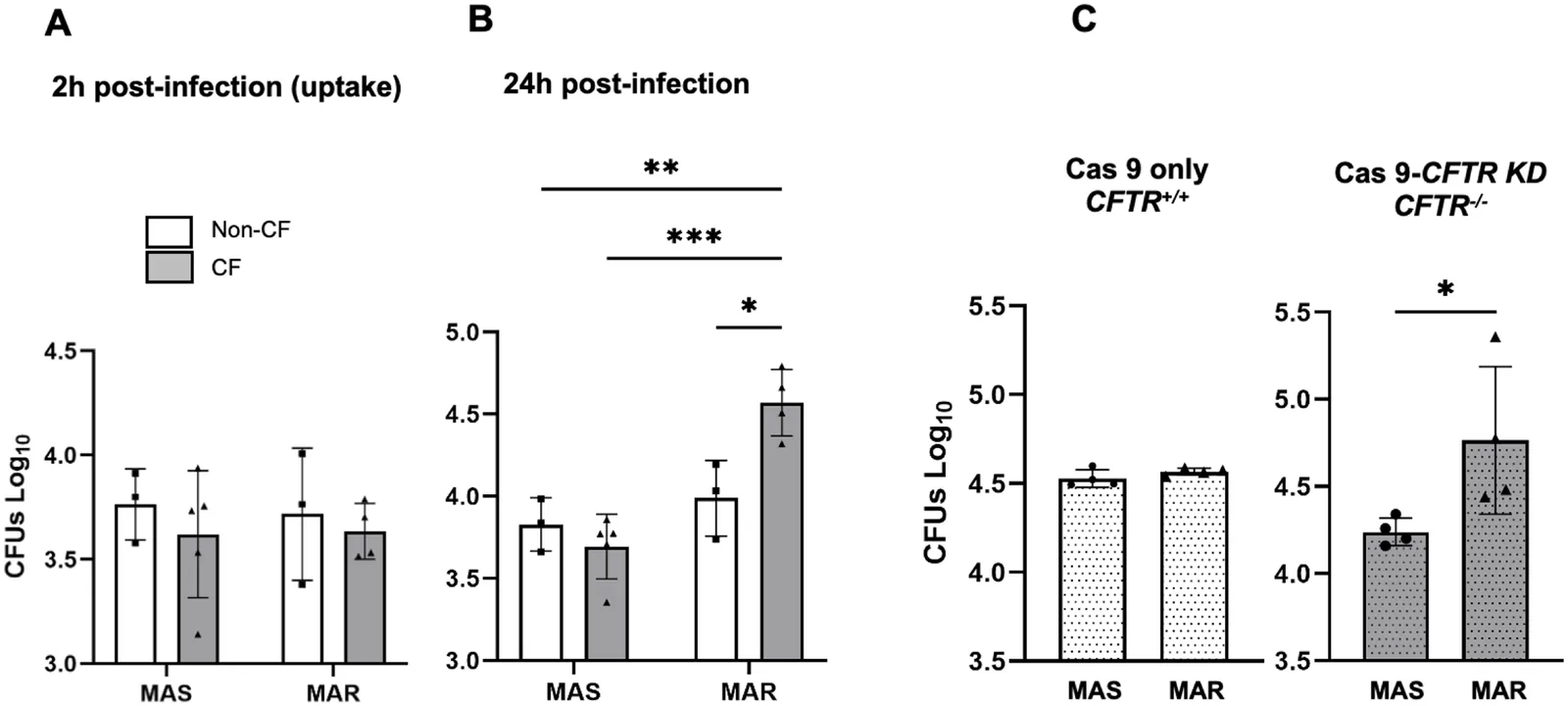
Enumeration of isolated MAB morphotypes in differentiated infected non-CF and CF macrophages. *MAB* smooth (*MAS*) or rough (*MAR*) morphotype infection in non-CF and CF monocyte-derived macrophages at 2h **(A)** and 24h post-infection **(B)** showing a significantly increased CF macrophage burden of *MAR* compared with MAR-infected non-CF macrophages and MAS-infected CF macrophages (***p=0.0007; **p=0.0092; *p=0.027), Tukey’s multiple comparisons test. N = 3 non-CF, N = 4 CF donors, in triplicate). Differentiated THP-1-like macrophages **(C)** treated with Cas9 only (CFTR^+/+^) showed no difference in intracellular *MAS* and *MAR* CFUs at 24h post-infection (p=0.5156), while Cas9+CFTR-specific guide RNA (CFTR knock down/CFTR^-/-^), recapitulated the increased burden of intracellular *MAR* relative to *MAS* at 24h post-infection (*p=0.0020) Wilcoxon t-test, N = 4 biological replicates each, with 3 technical replicates. Cas9 only (CFTR+/+) or Cas9+CFTR-specific guide RNA (CFTR knock down/CFTR-/-) showed similar levels of uptake at 2h post-infection (see **Supplementary Figure 1A**).

### *MAB* variants were resistant to killing by human serum and CF and non-CF macrophages and did not show differences in cytotoxicity with MAB infection

*MAB* variants were opsonized with human AB serum before infecting monocytes or macrophages to mimic conditions present in the lung (Ferguson et al., 2004). To determine if opsonization resulted in preferential killing of *MAB* morphotypes*, MAS* and *MAR* variants were incubated with no serum (DPBS control), 10%, or 50% human AB serum (**Supplementary Figure 2A**). Human serum significantly decreased the viability of *E. coli* (used as a positive control to evaluate killing) by 50% with 10% serum and to over 99% using 50% serum (p<0.0001), Two-way ANOVA. Human AB serum however failed to reduce viability of either *MAB* morphotype indicating that *MAB* was resistant to killing by human serum and that neither *MAS* nor *MAR* variant was significantly altered with opsonization. The increase in *MAR* in CF macrophages was not due to killing of the *MAS* variant prior to infection by CF macrophages.

We next determined whether *MAB* was cytotoxic to macrophages and might account for the difference in intracellular viability of *MAB* variants at 24h post-infection observed in CF and non-CF macrophages. Macrophage cytotoxicity was assessed by measuring LDH, a cytosolic enzyme that is released into the cell culture medium upon damage to cell plasma membranes. No significant difference in viability was observed among uninfected, *MAS*-infected, or *MAR*-infected CF and non-CF macrophages (p=0.8512), Two-way ANOVA (**Supplementary Figure 2B**), indicating that CF macrophages were not more susceptible to cell death by *MAB* relative to non-CF macrophages. N = 3 individual donors in duplicate for each group.

### Reactive oxygen species induction by *MAB* morphotypes was diminished in monocytes and monocyte-derived macrophages

Previous studies indicated extracellular *MAR* was more resistant than *MAS* to hydrogen peroxide (H_2_O_2_) *in vitro* (Clary et al., 2018). We hypothesized that suboptimal NADPH oxidase (NOX2) activation and generation of ROS might impact *MAR* killing in CF macrophages. Non-CF and CF monocytes were first examined, and the maximum ROS production was measured using luminol. PMA treatment was used as a standard agonist to stimulate ROS production via activation of protein kinase C (PKC). Un-opsonized *MAS-* or *MAR-*infected monocytes and uninfected monocytes generated little ROS in either non-CF or CF monocytes and macrophages (data not shown). PMA-stimulated non-CF monocytes produced a defined peak depicting the maximum ROS burst (**Supplementary Figure 2A**) and healthy monocytes infected with *MAS* (p=0.0002) or with *MAR* (p<0.0001) exhibited a significantly diminished ROS burst, relative to uninfected PMA-treated healthy monocytes, (N = 8 donors), ordinary one-way ANOVA with multiple comparisons (**Supplementary Figures 2A, C**). Notably uninfected CF monocytes produced a significantly diminished ROS burst relative to non-CF monocytes (p=0.0174) and CF monocytes also showed significantly less ROS when infected with *MAS* (p<0.0001) or *MAR* (p=0.0005) relative to PMA-treated non-CF monocytes, (N = 10 donors) (**Supplementary Figures 2B, C**). *MAS-* and *MAR*-infected CF monocytes were not statistically different from uninfected PMA-treated CF monocytes. AUC analyses were normalized to PMA-treated healthy controls after subtracting background from untreated/uninfected monocytes.

The DCF assay was used to investigate ROS production in CF and non-CF monocyte-derived macrophages, as described (Assani et al., 2017). Representative time course ROS burst experiments are shown for non-CF macrophages (**Figure 2A**), CF macrophages (**Figure 2B**), and the combined AUC ROS burst data of non-CF (N = 6) and CF donors (N = 4) (**Figure 2D**) were normalized to PMA-treated controls as for monocytes after subtracting the background signal from untreated/uninfected macrophages. To further investigate the contribution of CFTR function to diminished ROS production during infection, we pharmacologically inhibited healthy macrophages with INH-172, which impedes CFTR-mediated ion conduction and channel gating (Young et al., 2024). The AUC of each burst assay of healthy non-CF macrophages (N = 6) and healthy non-CF macrophages treated with 5μM INH-172 (N = 4) (**Figures 2C, D**) mirrored results observed with CF macrophages. Relative to healthy PMA-treated uninfected macrophages, *MAR*-infected CF macrophages exhibited diminished ROS production (p=0.0418) and CFTR inhibitor-treated *MAR*-infected healthy macrophages also showed a significantly decreased ROS burst (p=0.0497) relative to uninfected PMA-treated macrophages without INH-172 treatment, ordinary one-way ANOVA with multiple comparisons. The change in ROS production in CF *MAS*-infected macrophages and in CFTR inhibitor treated non-CF macrophages were not statistically different relative to non-CF PMA-treated uninfected macrophages.

**Figure 2:**
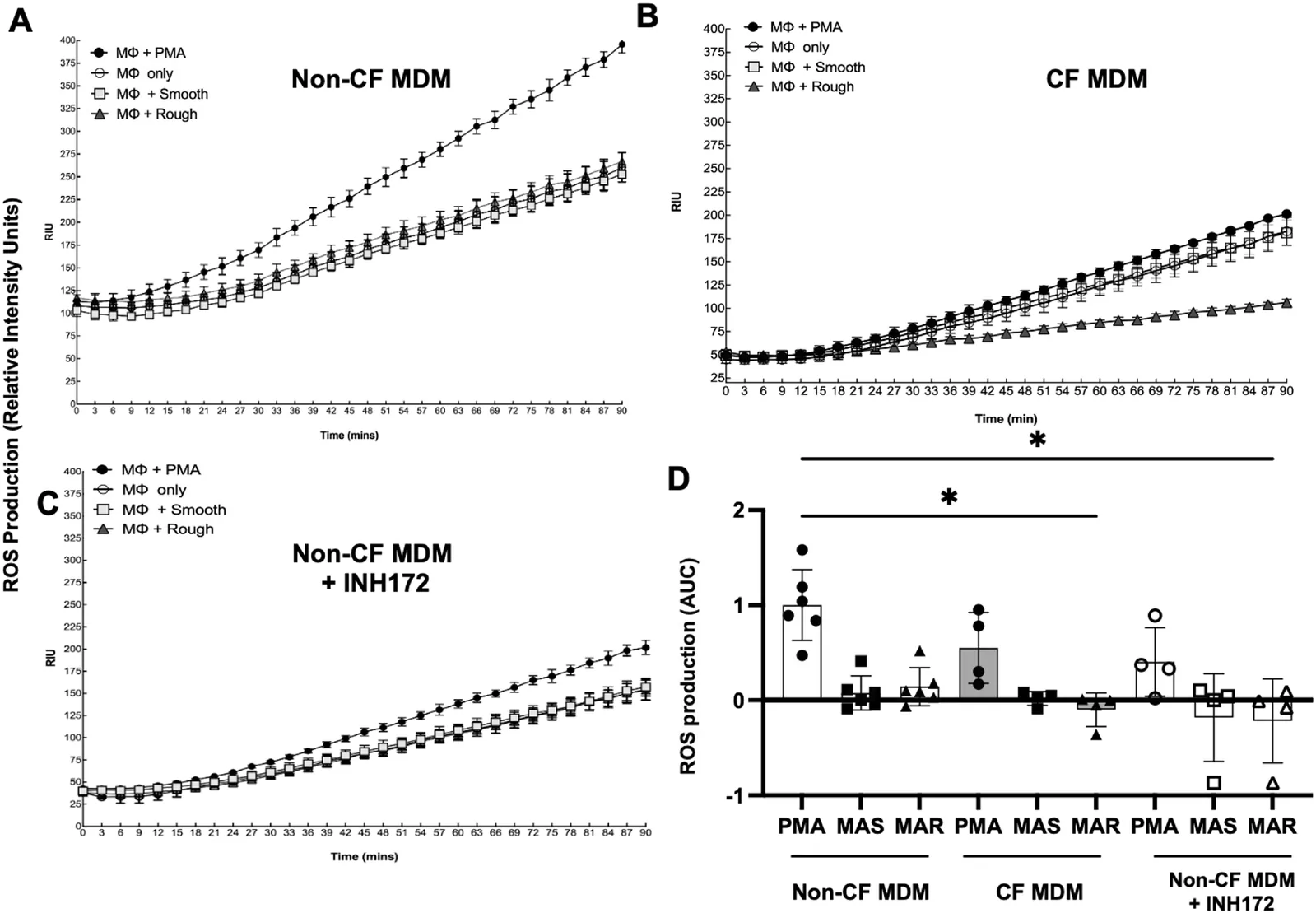
Macrophage ROS Burst Assays. Representative time course ROS burst experiment for non-CF macrophages **(A)**, CF macrophages **(B)**, and non-CF macrophages **(C)** measured by DCF. The combined AUC ROS burst (D) of non-CF (white bars, N=6), CF macrophages (gray bars, N=4), and non-CF macrophages + INH-172 were normalized to PMA-treated controls as for monocytes after subtracting the background signal from untreated/uninfected macrophages (circles). Relative to healthy PMA-treated macrophages, *MAR*-infected CF macrophages (triangles) (*p=0.0418) and *MAR*-infected INH-172-treated non-CF macrophages (*p=0.0497) elicited significantly reduced ROS production. Unstimulated controls are shown as (open circles). All AUC analyses were normalized to PMA-treated healthy controls after subtracting background from untreated/uninfected monocytes or macrophages and analyzed by ordinary one-way ANOVA with Kruskal -Wallis test followed by Dunn's multiple-comparisons test.

For comparison, alveolar macrophages obtained from bronchial alveolar lavage (BAL) from one CF donor were evaluated by the DCF assay. A time course experiment of a CF alveolar macrophage burst assay is shown in **Supplementary Figure 4A**. CF alveolar macrophages showed a similar trend of reduced ROS production in *MAB*-infected alveolar macrophages shown in **Supplementary Figure 4B**, depicting the AUC of PMA-treated, *MAS*-infected, and *MAR*-infected alveolar macrophages after subtracting the background signal of untreated, uninfected macrophages. Together, these data suggest that CFTR dysfunction may contribute to the impaired response of CF macrophages to *MAB* infection.

### CF macrophages showed diminished phosphorylation of NOX2 p47^phox^ relative to non-CF macrophages following infection with *MAB*

Macrophage activation and phosphorylation results in recruitment of NADPH Oxidase 2 (NOX2) components p47^phox^ and p40^phox^ from the cytoplasm to form a complex with membrane bound gp91. The reduced ROS production in CF macrophages and in non-CF macrophages treated with a CFTR inhibitor suggested that *MAB* infection may impact phosphorylation of NOX2 components. We evaluated total expression and phosphorylation of p47^phox^ and p40^phox^ as well as total membrane-bound gp91 in non-CF and CF macrophages by western blot normalized to GAPDH at 2h and 24h post-infection and compared to uninfected macrophages for each experiment (**Figures 3A–C**). Although there was considerable donor-donor variability, aggregate data of combined AUC analyses indicated that CF macrophages exhibited reduced phosphorylation of p47^phox^ post-infection relative to non-CF macrophages (N = 6 healthy controls, N = 5 CF donors). At 2h post-infection, CF macrophages showed 31.2% phosphorylation of p47^phox^ infected with *MAS* and 63.2% p47^phox^ phosphorylation infected with *MAR*, relative to non-CF macrophages. At 24h post-infection however, p47^phox^ phosphorylation was significantly decreased in CF macrophages (p=0.0199) compared with non-CF macrophages, two-way ANOVA. No difference was observed between CF and non-CF macrophages infected with *MAS*. Calculation of the ratios of individual relative band intensities for phosphorylated/total p47 and p40 confirmed these results (see **Supplementary Figure 5**).

**Figure 3:**
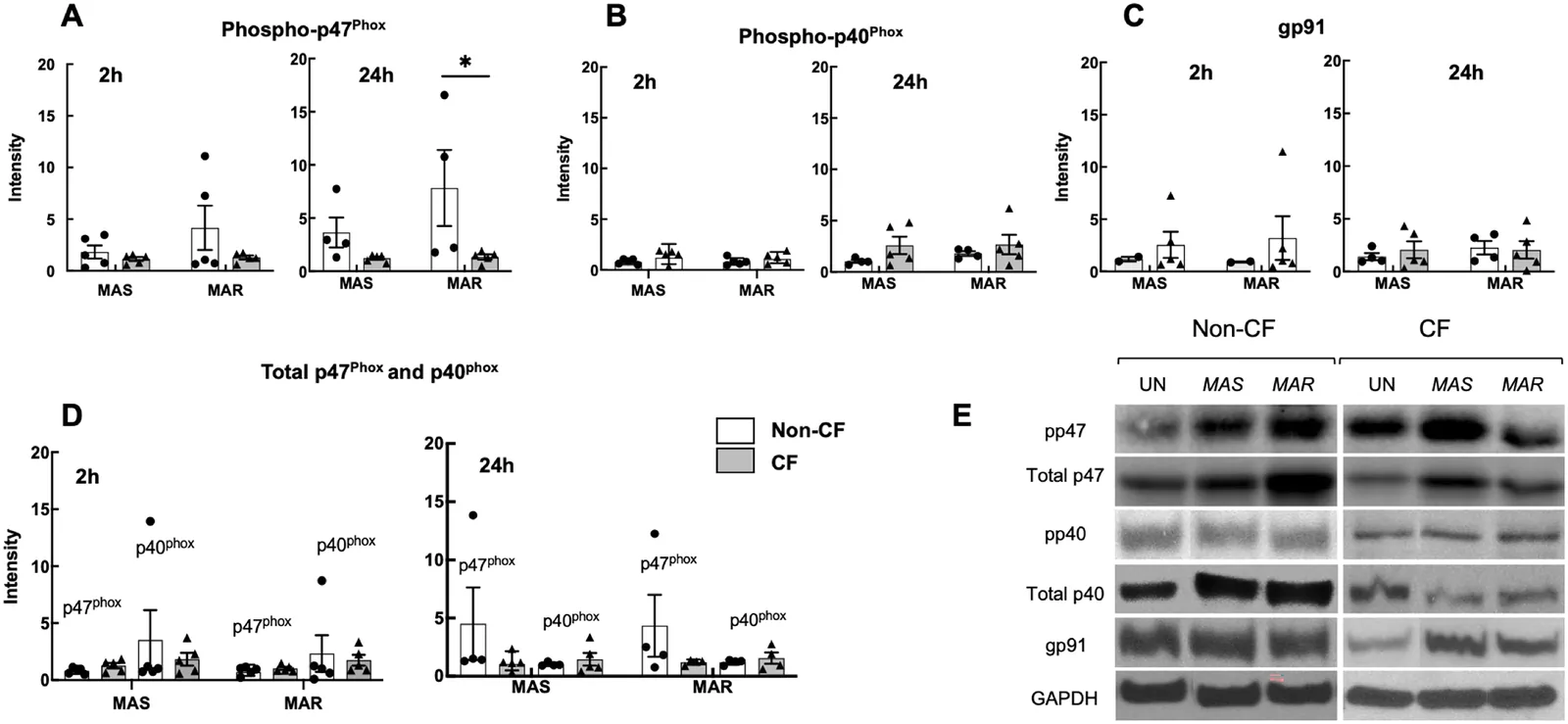
NOX2 protein analysis for *MAB*-infected CF and non-CF monocytes and macrophages. Western blot densitometry analysis of NOX2 components in non-CF MDM and CF MDM at 2 and 24h post-infection **(A–D)** show that phospho-p47^phox^ was reduced at 2h and significantly decreased in CF macrophages relative to non-CF macrophages (p=0.0199) at 24h post-infection **(A)**. There were no differences in phosphorylation of other NOX2 components **(B, C)** or in total protein between CF and non-CF macrophages **(D)**. Non-CF donors (N = 4) per condition, normalized to GAPDH and CF donors (N = 5) per condition, normalized to GAPDH. Representative images of non-CF and CF macrophage protein lysates of NOX2 components are shown **(E)**. Calculated ratios of NOX2 proteins showed comparable results * p=0.0199 (see **Supplementary Figure 5**).

Since PMA activation of protein kinase C (PKC) stimulates migration of NOX2 components from the cytoplasm to associate with membrane bound NOX2 components, we also evaluated whether dysregulated PKC isoform phosphorylation impacted NOX2 assembly and the optimal production of the oxidative burst (Assani et al., 2017; Parihar et al., 2018). Western blot analysis indicated that expression and phosphorylation of PKC isoforms was not significantly different between healthy and CF macrophages with *MAR* or *MAS* infection. N = 5 donors each non-CF and CF for pPKCα β and pPKCδ; N = 3 each for pPKCζ/γ, **Supplementary Figures 6A–C**.

### *MAB*-infection altered the inflammatory cytokine profile in CF macrophages

CF macrophages produce exaggerated pro-inflammatory cytokines compared to non-CF macrophages in response to many CF pathogens, suggesting that CF macrophages are hyperinflammatory (Kopp et al., 2012; Bruscia and Bonfield, 2016; Assani et al., 2017). Cytokine production in uninfected macrophages from healthy and CF donors were not different (**Supplementary Figure 7**), however *MAB*-infected CF and non-CF macrophages exhibited a distinct pattern of pro-inflammatory cytokine production (**Figure 4**). IL-8 (CXCL8) production was significantly increased in CF macrophages relative to non-CF macrophages upon infection with *MAB* regardless of bacterial morphotype (p<0.0001), N = 5 donors each. *MAS*-infected CF macrophages produced significantly more MIP-1β (p=0.0216) and IL-6 (p=0.0289) compared with non-CF macrophages (**Figures 4B, C**), however there was no difference between healthy and CF macrophages infected with *MAR*. In contrast, TNF-α production was significantly decreased in CF macrophages infected with *MAR* relative to *MAR*-infected non-CF macrophages (p=0.0072), (**Figure 4D**). There was no difference in TNF-α production between CF and non-CF macrophages infected with *MAS*. Interestingly IL-1β secretion was significantly lower in *MAB*-infected CF macrophages compared with non-CF macrophages (p<0.0001, N = 4 non-CF, N = 4 CF donors) (**Figure 4E**) compared with IL-1β production in previous reports (Zhang et al., 2020; Jaganathan et al., 2022) and suggested that *MAB* suppressed the IL-1β response.

**Figure 4:**
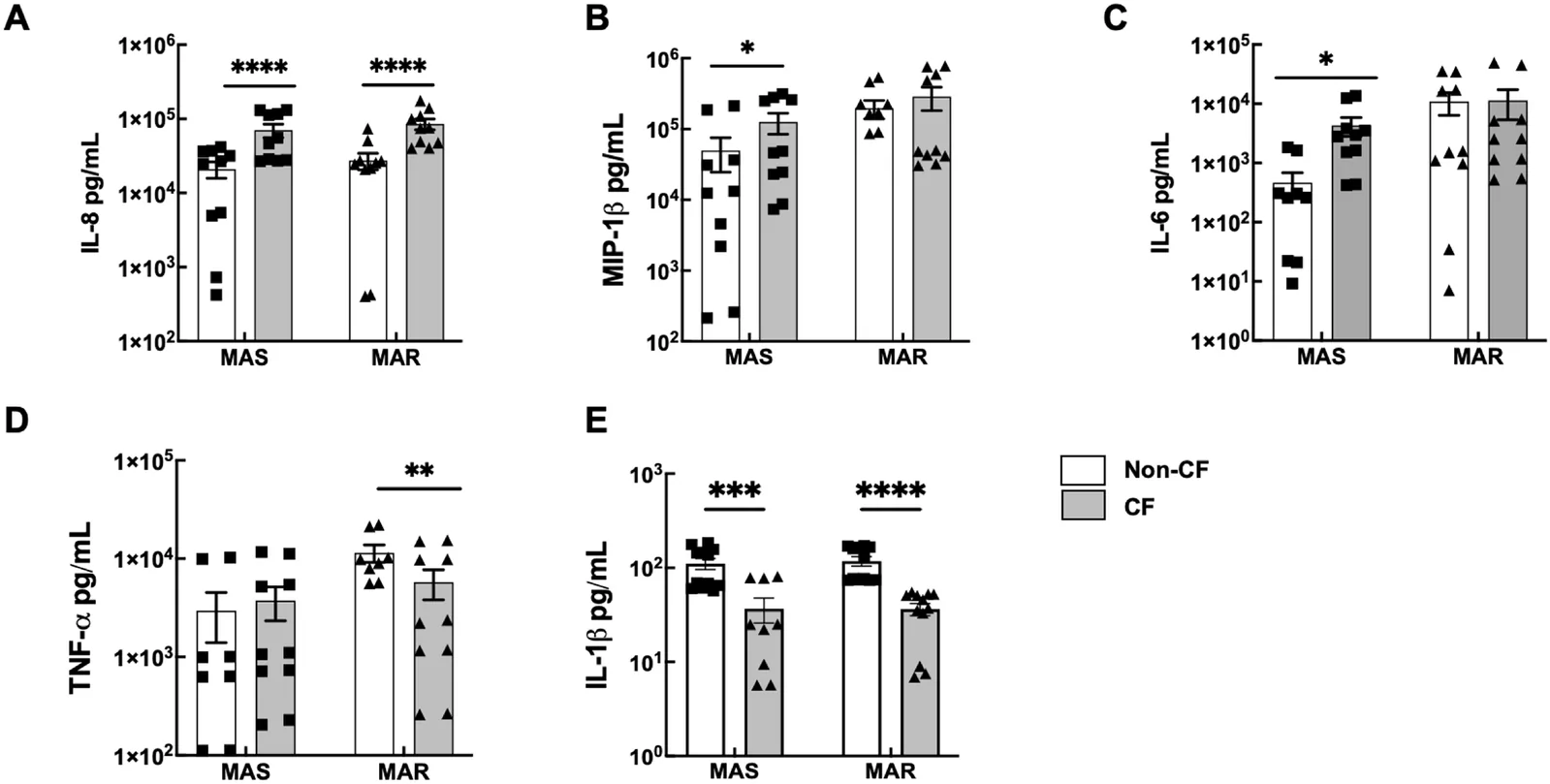
Pro-inflammatory cytokine production by *MAB*-infected non-CF and CF macrophage at 24h post-infection. Interleukin 8 (IL-8) **(A)** ****p<0.0001 (N=5 each non-CF, and CF donors, in duplicate); Macrophage inflammatory protein 1beta (MIP-1b **(B)** *p=0.0216 (N=5 each non-CF, and CF donors, in duplicate); Interleukin 6 (IL-6), *p=0.0289 **(C)**; Tumor Necrosis Factor alpha (TNF-a) **(D)** **p=0.0072 (Non-CF N=4, CF N=5, in duplicate; and Interleukin 1 beta (IL-1b) ***p=0.0001, ****p<0.0001 (Non-CF, CF N=4, in triplicate **(E)**. Non-CF (white bars), CF (gray bars), MAS (squares) MAR (triangles). Two-way ANOVA. Uninfected controls are shown in **Supplementary Figure S7** and showed no significant difference between uninfected levels of proinflammatory cytokines between non-CF and CF macrophages.

We hypothesized that reduced secretion of IL-1β resulted from defective production of mature IL-1β since pro-IL-1β is cleaved by caspase 1. Both proteins were evaluated by Western blotting. Following normalization to GAPDH, no significant difference in pro-IL-1β levels was observed between *MAB*-infected healthy and CF macrophages. Similarly, pro-caspase 1 did not differ between the two groups (N = 3 non-CF and CF each) (**Figures 5A–C**). However, no mature IL-1β or caspase-1 protein was detected in infected macrophages at 24h (**Figure 5C**), consistent with the very low levels of secreted IL-1β observed in healthy or CF macrophages infected with *MAB*.

**Figure 5:**
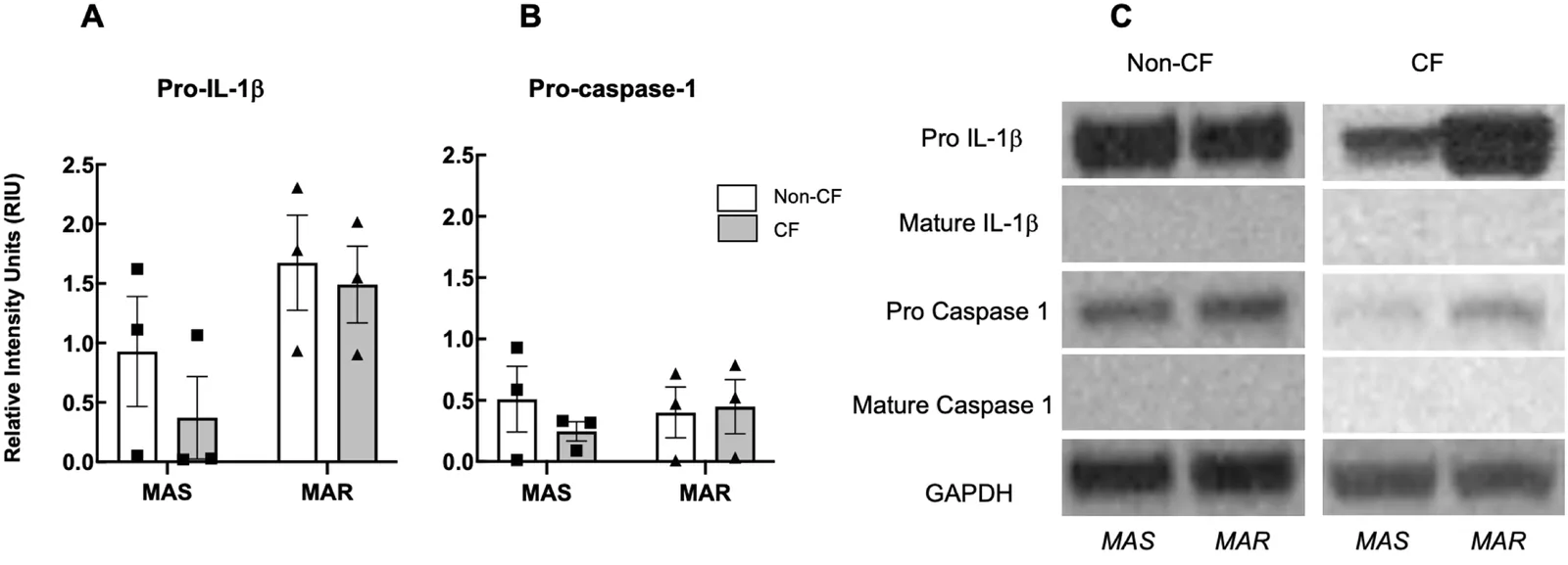
IL-β and caspase-1 protein analysis. There was no difference in pro-IL-1β or pro-caspase 1 between groups (normalized to GAPDH). No mature IL-1β or caspase-1 protein was detected in infected macrophages at 24h, aligned with the very low levels of secreted IL-1β by both healthy and CF macrophages infected with *MAB*. Non-CF and CF (N = 3) each, Two-way ANOVA.

The low levels of IL-1β and decreased TNF-α in CF *MAR*-infected macrophages suggested that *MAB* infection might interfere with apoptosis. Apoptosis, measured by Annexin V, was not significantly different between uninfected non-CF and CF macrophages (p= 0.3648) (**Figure 6**). While there was an increase overall in apoptosis in *MAS*-infected macrophages relative to uninfected macrophages, apoptosis in *MAS*-infected non-CF and CF macrophages was not significantly different (p=0. 0.8703). In contrast, *MAR*-infected CF macrophages exhibited significantly reduced apoptosis compared with non-CF macrophages (p=0.0321). Notably, while there was no difference in the percent of apoptotic cells between *MAS*- and *MAR*-infected non-CF macrophages (p=0.7423), apoptosis was also significantly reduced in CF *MAR*-infected relative to *MAS*-infected macrophages (p=0.0127), paired t test (**Figure 6**). Confocal microscopic images of Annexin V staining for apoptosis (**Supplementary Figure 8**) show representative low levels of apoptosis in infected macrophages.

**Figure 6:**
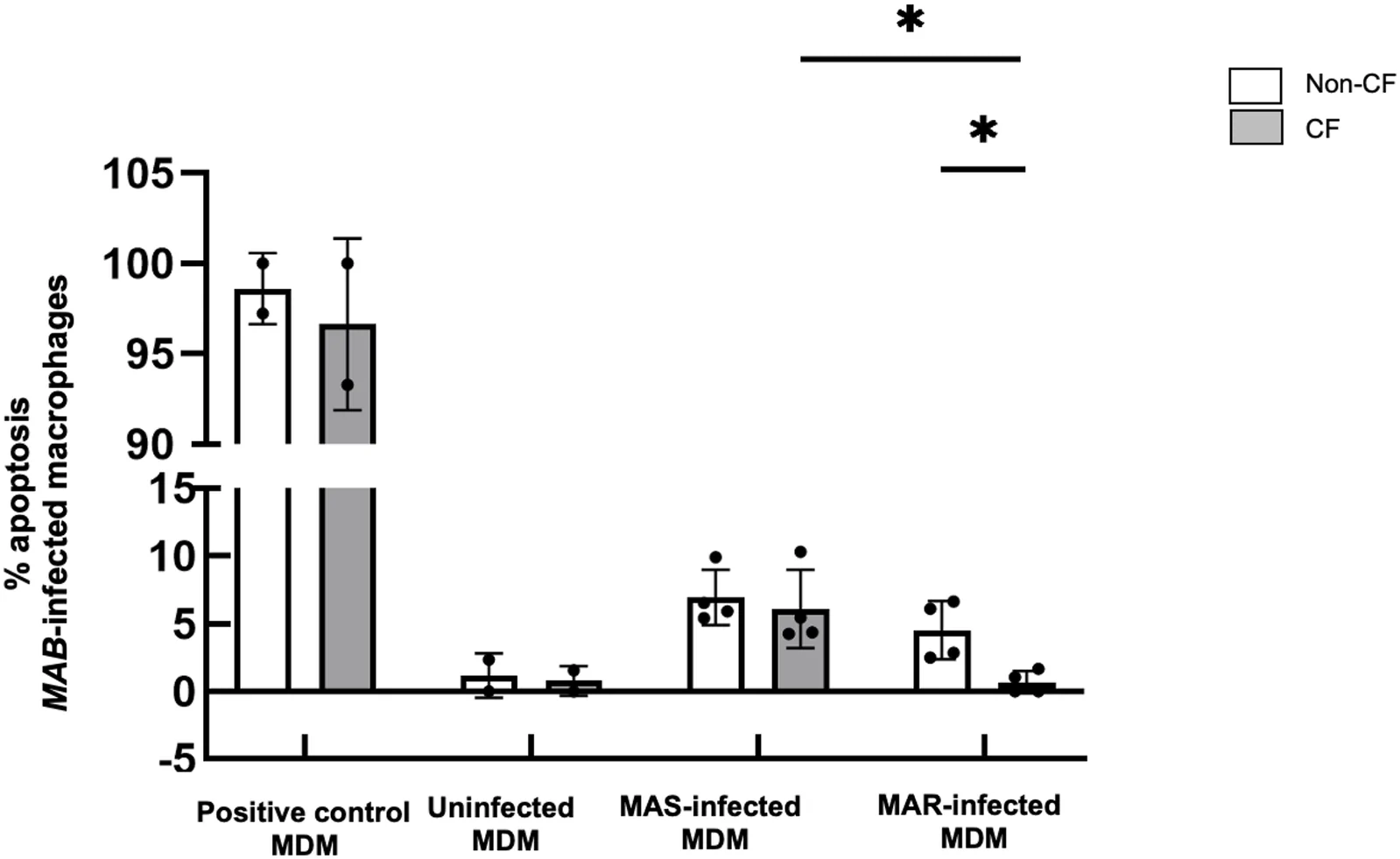
Percent apoptosis in non-CF and CF macrophages 24h post-infection. Both positive controls and uninfected non-CF and CF macrophages showed no difference in apoptosis (p= 0.3648) between non-CF and CF macrophages. Moreover, uninfected non-CF and CF macrophages showed low levels of apoptotic macrophages. While the percent of apoptosis increased in MAS-infected macrophages compared with uninfected macrophages, non-CF and CF macrophages did not show significantly different levels of apoptosis (p=0.8703) and there was no difference in the percent of apoptotic macrophages between MAS and MAR in non-CF macrophages (p=0.8398). In contrast, MAR-infected CF macrophages evidenced significantly reduced apoptosis compared with both non-CF macrophages (*p=0.0321), paired t test, and relative to MAS-infected CF macrophages (*p= 0.0127). Non-CF (white bars), CF (gray bars). N=3 independent donors each non-CF and CF, in triplicate; apoptotic cells were enumerated per 100 macrophages for each sample.

## Discussion

Macrophages are critical for bacterial eradication in the lung, and CFTR dysfunction leads to defective microbial clearance by innate immune cells that can contribute to persistent airway infections and dysregulated inflammation in pwCF (Bruscia et al., 2009; Bruscia and Bonfield, 2016; Assani et al., 2017; Jaganathan et al., 2022). Activated monocytes are recruited to the lungs of pwCF and monocyte-derived macrophages are significantly elevated compared with a non-CF cohort (Schupp et al., 2020). In previous studies we showed that *MAB* variants survived for up to 48h in differentiated THP-1 macrophages, even with antibiotic treatment (Clary et al., 2018) suggesting that macrophages can serve as a safe harbor for *MAB*. In the current study we used primary human monocytes and differentiated monocyte-derived macrophages from CF and non-CF donors as a relevant model of lung macrophage function.

Macrophage uptake of *MAB* variants at 2h post-infection did not differ between groups. In non-CF macrophages, intracellular *MAS* and *MAR* at 24h were comparable, suggesting that healthy macrophages limited growth of both *MAB* morphotypes without achieving full eradication. Strikingly, CF macrophages limited *MAS* similarly to healthy macrophages but harbored a significantly higher burden of intracellular *MAR* at 24h, indicating a morphotype-specific impairment in CF macrophage function. CFTR knockdown in THP-1 derived macrophages recapitulated significantly increased intracellular *MAR* at 24h post-infection, without affecting viable intracellular *MAS* and supported a functional role for CFTR in limiting intracellular *MAR*.

CF monocytes and macrophages treated with PMA to induce ROS showed significantly reduced ROS production, and *MAB* infection further suppressed ROS in both CF and non-CF cells. However, the rough *MAR* variant suppressed ROS in CF macrophages and in non-CF healthy macrophages treated with the CFTR inhibitor. Impaired NOX2 activation was observed in *MAR*-infected CF macrophages shown by decreased phosphorylated p47^phox^ at 2h and a significant decrease at 24h post-infection compared to healthy macrophages, while other NOX2 components and PKC phosphorylation did not differ. These findings are consistent with the known hyper-virulence of rough *MAB* (Catherinot et al., 2009; Hedin et al., 2023). These results agree with prior work from our group showing CFTR-dependent NOX2 impairment in *Burkholderia cenocepacia*-infected macrophages (Kopp et al., 2012; Assani et al., 2017; Jaganathan et al., 2022). Diminished phosphorylated p47^phox^ in *B. cenocepacia*-infected CF macrophages was recapitulated with CRISPR-Cas CFTR-knockout macrophages and showed reduced oxidative burst, increased bacterial load, and increased apoptosis (Zhang et al., 2023). Studies recently demonstrated an essential role of CFTR in neutrophil antibacterial function (Robledo-Avila et al., 2026). CFTR knockout was also shown to impair NOX-mediated bactericidal activity in a zebrafish *MAB* model and supports a role for CFTR in decreased neutrophil recruitment and the formation of protective granulomas that resulted in hyper-susceptibility to infection and larval death with both morphotypes (Bernut et al., 2019). Our results suggest that the exacerbated reduction of ROS in *MAR*-infected CF macrophages may contribute to the ability of CF macrophages to limit intracellular *MAR* growth. While not studying different morphotypes, a recent study also found that CF macrophages were unsuccessful in controlling intracellular *MAB* and that CFTR dysfunction was associated with decreased mitochondrial ROS production (Tarique et al., 2026).

Notably *MAB*-infected CF macrophages exhibited a distinct pro-inflammatory cytokine profile. Previous studies from our group focused on CFTR dysfunction in *B. cenocepacia*-infected macrophages showed that CF macrophages produced high levels of pro-inflammatory cytokines including TNF-α, IL-6, MIP-1β (CCL4), and IL-8 (CXCL8) relative to non-CF macrophages (Kopp et al., 2012; Assani et al., 2017). *MAB-*infected CF macrophages produced more IL-8 compared with healthy uninfected macrophages with either *MAB* morphotype, consistent with the intense neutrophilic infiltration characteristic of NTM infection in a murine model and in pwCF (Caverly et al., 2015; Hayes et al., 2022). Interestingly, while *MAS*-infected CF macrophages produced significantly more MIP-1β and IL-6 compared with healthy macrophages indicating a robust pro-inflammatory cytokine response, *MAR*-infected CF macrophages showed no difference in these cytokines compared to healthy macrophages. Remarkably, both *MAB* morphotypes suppressed IL-1β secretion significantly in CF macrophages compared to non-CF macrophages at 24h post-infection. IL-1β secretion typically follows caspase-1 activation and cleavage of pro-IL-1β to produce mature IL-1β, which is secreted by macrophages. No mature IL-1β or cleaved caspase-1 was detectable at 24h despite comparable pro-IL-1β and caspase-1 protein levels. These results indicate that both *MAB* morphotypes disrupted IL-1β secretion. *Mycobacteria tuberculosis* infection has also been shown to disrupt IL-1β secretion by preventing inflammasome assembly, activation, and IL-1β processing (Master et al., 2008) and exogenous IL-1β was shown to restrict replication of *M. tuberculosis* in macrophages (Jayaraman et al., 2013).

TNF-α production was also significantly reduced in *MAR*-infected CF macrophages relative to healthy non-CF macrophages, while *MAS*-infection produced comparable TNF-α levels in CF and non-CF macrophages. This was surprising since *MAR* is widely considered to be more inflammatory than *MAS* (Rhoades et al., 2009; Floto et al., 2016) and TNF-α was significantly increased in CF macrophages infected with *B. cenocepacia* (Kopp et al., 2012; Zhang et al., 2020). Several reports however suggest that TNF-α plays a key role in mycobacterial infections. Clinically, TNF-α inhibitors are linked to an elevated risk of NTM infection (Brode et al., 2015) and a reported tumor necrosis factor receptor associated factor 3 (TRAF3) mutation in an adult with recurrent bronchiectasis and pulmonary *MAB* infection exhibited both diminished TRAF expression and TNF-α secretion (Liew et al., 2022). IL-1b enhanced TNF-a-mediated inflammatory responses (Saperstein et. al, 2009). Mechanistic evidence has shown that IL-1β-induced macrophage killing of *M. tuberculosis* was mediated by TNF signaling, increased TNF secretion, and TNFR1 expression (Jayaraman et al., 2013) and mutation of *M. tuberculosis* type I NADH dehydrogenase that neutralizes host ROS production, resulted in increased macrophage TNF-α secretion and apoptosis, and restricted mycobacterial survival (Miller et al., 2010). The established role of TNF-α in apoptosis (van Loo and Bertrand, 2023) as well as reduced apoptosis in CFTR KO MDM, led us to hypothesize that *MAB-*infection might limit macrophage cell death in CF macrophages. Indeed, *MAR*-infected CF macrophages showed significantly fewer apoptotic macrophages compared with *MAR*-infected healthy macrophages and *MAS*-infected CF macrophages, while no difference was detected between uninfected or between *MAS*-infected groups. It is unclear if CFTR-KO is associated with inflammatory cytokine production (Zhang et al., 2023) however these results suggest that CFTR alone may not be responsible for the failure of CF macrophages to limit intracellular M*AR*.

While recapitulating many aspects of human differentiated monocyte-derived macrophages, our study had limitations. First, since CF BAL macrophages are extremely rare, we were only able to obtain one sample. However, CF alveolar macrophages showed lower ROS production compared with PMA, and was supportive of data with CF MDM. Second, we did not test to see if ETI treatment rescued some of the described defects in CF macrophages. Studies with *MAB* are part of an ongoing study on pathogen responses to ETI, however our group has previously shown that ETI is associated with select changes in innate immune function in macrophages and neutrophils (Zhang et al., 2023; Robledo-Avila et al., 2026). Moreover, while not studying different morphotypes, a recent study also using CF MDM found that control of *MAB* was only partially improved by ETI (Tarique et al., 2026).

In the present study we show that although *MAB* was not fully eradicated in both CF and healthy macrophages, CF macrophages showed a significant and profound impaired response to *MAR* infection. Our results suggest that *MAR*-infected CF macrophages exhibited compounding defects including impaired macrophage killing of *MAR*, reduced ROS production and p47^phox^ activation, diminished TNF-α secretion, and decreased apoptosis. Together with the impaired ROS production and global *MAB*-induced suppression of IL-1β in CF macrophages, deficient TNF-α signaling in *MAR*-infected CF macrophages may have further suppressed apoptosis, which created conditions that were permissive for *MAR* persistence. These findings suggest that *MAR* may share immune evasion strategies with *M. tuberculosis* to subvert macrophage activation and cytokine signaling. Our results complement zebrafish studies showing that CFTR depletion or loss of TNFR1 reduced ROS production and markedly increased susceptibility to *MAB* infection (Bernut et al., 2016, 2019). Overall, *MAB* demonstrated features of a stealth intracellular pathogen that persists in human macrophages. Our results further indicate that CF macrophages were acutely vulnerable to the virulent rough *MAB* morphotype and support including consideration of colony morphology in the clinical management of *MAB* infection in pwCF.

## Data Availability

The raw data supporting the conclusions of this article will be made available by the authors, without undue reservation.

## Supplementary material

Supplementary Figure 1 Differentiated THP-1-like macrophages treated with Cas9 only (CFTR^+/+^) or Cas9+CFTR-specific guide RNA (CFTR knock down/CFTR^-/-^) showed similar levels of uptake at 2h post-infection (A). THP-1 CFTR clones, depicted in (B), show that Clone 10F8 used for infection had the greatest reduction in CFTR compared to homogenous Cas9-sgCFTR THP-1 cells. CFTR KD THP-1 after 1 and 2 dox treatments (C). Lane 1 was a scrambled control, showing that CFTR is not affected. Lane 2 (+1dox ), and Lane 3 (+2dox ), showed maximum reduction in CFTR, whereas Lane 4 (no dox control) showed CFTR bands.

Supplementary Figure 2 *MAB* resistance to human serum and viability of infected macrophages. *MAS* and *MAR* morphotypes resisted killing by human AB serum (ABS) (A). *MAB* morphotypes were not susceptible to serum killing. PBS buffer alone control (black bars); 10% serum (light gray bars); 50% serum (dark gray). *E. coli* in contrast was susceptible to serum killing in a dose dependent manner. N = 3 biological replicates, p<0.0001 for *E. coli*, *MAR* and *MAS* viability was not significantly different from PBS control. (B). *MAB-*infected macrophage viability. *MAB*-infected macrophages from non-CF controls (white bars) or from pwCF (gray bars) were not significantly different from uninfected macrophages among groups. N = 3 individual donors averaged duplicates for each group, Two-way ANOVA.

Supplementary Figure 3 Monocyte ROS Burst Assays. Representative time course monocyte experiment showing the non-CF monocyte ROS burst **(A)** and the CF monocyte ROS burst **(B)** measured by luminol relative intensity units (RIU). The combined area under the curve (AUC) **(C)** of each burst assay of of uninfected (circles) non-CF (white bars), and CF donors (gray bars) is depicted relative to the maximal ROS burst exhibited by non-CF PMA-treated uninfected monocytes. Non-CF (N=8) and CF (N=10). Compared to uninfected PMA-treated healthy monocytes, non-CF monocytes exhibited significantly reduced ROS with MAS (****p<0.0001) and MAR infection (***p=0.0002). CF PMA-treated monocytes showed a reduced burst (*p=0.0174) relative to healthy monocytes suggesting reduced ROS capability. However, CF MAS-infected (****p<0.0001) and MAR-infected (***p=0.0005) monocytes were similarly impaired in ROS production relative to healthy monocytes. Uninfected (circles), MAS (squares), MAR (triangles), ordinary one-way ANOVA with multiple comparisons.

Supplementary Figure 4 ROS burst of CF bronchoalveolar macrophages. Bronchoalveolar macrophages (AM) (N = 1) infected with *MAS* or *MAR* (A) measured by (relative Intensity Units) (RIU) are depicted in the AUC (B) and support data showing that ROS production is reduced with *MAB* infection. (N = 1 CF donor). The bronchoalveolar sample was centrifuged and washed to remove mucus and debris and washed before attaching to tissue culture plates overnight. Non-adherent cells were washed and infected by *MAS* or *MAR* as for MDM.

Supplementary Figure 5 Calculated ratios of NOX2 protein analysis for MAB-infected CF and non-CF macrophages. Western blot densitometry analysis of NOX2 components in non-CF MDM and CF MDM at 2 and 24 h post-infection (A–C) showed that calculated ratios of individual relative band intensities for P47 and P40 indicated that Phospho-p47phox was significantly decreased for CF macrophages relative to non-CF macrophages (*p=0.0199) at 24h post-infection (A) (comparable results to **Figure 3**). Non-CF donors (N = 4) per condition, normalized to GAPDH and CF donors (N = 5) per condition, normalized to GAPDH. Representative images of non-CF and CF MDM protein lysates of NOX2 components are shown (D).

Supplementary Figure 6 Phosphorylated Protein Kinase C in *MAB*-infected non-CF and CF Macrophages. Aggregate Western blot relative intensity unit (RIU) analysis of PKC isoforms in *MAS-*infected or *MAR-*infected non-CF and CF macrophage lysates at 24h post-infection. Phosphorylated PKC subclass expression, conventional: α/β, novel: δ (N=5), and atypical: ζ/γ) (N = 3) individual donors, for non-CF and CF each were normalized to GAPDH are shown in (A–C). Representative blots are depicted in (D).

Supplementary Figure 7 Uninfected Non-CF and CF macrophages cytokine production. Cytokine secretion by uninfected non-CF and CF macrophages was not significantly different: IL-8 (p=0.1533), MIP-1β (p=0.1481), IL-6 (p=0.3929), and TNF-α (p=0.4345). Data were analyzed using Welch’s unpaired t-test following log10 transformation. IL-1β was undetectable in uninfected macrophages.

Supplementary Figure 8 Representative microscopic images of Annexin V positive non-CF and CF macrophages. Representative confocal microscopic images showing non-CF (A-C) and CF MAB-infected (D-F). Apoptosis was measured by Annexin V fluorescence and compared with a positive control (induced apoptosis by RNase treatment in uninfected macrophages) for each group. Annexin V positive cells are shown in green. Arrows indicate mCherry *MAS*- or *MAR*-infected macrophages.
